# Modeling gliomas with organoids: reconstructing the human neural microenvironment for translational neuro-oncology

**DOI:** 10.3389/fonc.2026.1803705

**Published:** 2026-05-15

**Authors:** Mohamed Ishan, Jorge Luis Jimenez-Macias, Renee D. Read

**Affiliations:** 1 Department of Pharmacology and Chemical Biology, Emory University School of Medicine, Atlanta, GA, United States; 2 Department of Hematology and Medical Oncology, Emory University School of Medicine, Atlanta, GA, United States; 3 Winship Cancer Institute, Emory University School of Medicine, Atlanta, GA, United States

**Keywords:** brain organoids, cancer therapy, glioblastoma, glioma, tumor invasion, tumor microenvironment, tumor synapse, glioma organoids

## Abstract

Gliomas represent some of the most lethal and biologically complex tumors of the central nervous system, with poor outcomes across both adult and pediatric populations. Beyond tumor-intrinsic genetic alterations, glioma progression is increasingly recognized to be driven by dynamic interactions with the neural microenvironment, particularly through direct tumor–neuron communication. Studies of adult and pediatric gliomas, including glioblastoma (GBM) and diffuse midline glioma (DMG), using single-cell profiling and multi-omics technologies have revealed extensive tumor intrinsic and extrinsic heterogeneity, with phenotypically and genetically plastic tumor cells actively engaged with neurons, glia, vasculature, and immune compartments that collectively reshape the neural microenvironment to promote tumor growth, invasion, and therapy resistance. Conventional two-dimensional *in vitro* culture systems and *in vivo* animal models incompletely recapitulate these interactions, limiting mechanistic insights and constraining clinical translation to human patients. Recent advances in organoid technologies have addressed this gap, enabling the development of three-dimensional human-specific models of glioma-microenvironment interactions. Platforms such as iPSC-derived cerebral organoids, regionally patterned neural organoids, patient-derived glioma organoids and tumor–organoid co-culture systems capture essential features of these human diseases. These systems recapitulate tumor–neuron synaptic integration, diffuse invasion programs, activity-dependent tumor growth, hypoxia-structured niches, vascular interactions, and patient-specific therapeutic responses. In this review, we synthesize recent advances and biological insights gleaned across glioma organoid model systems and evaluate their strengths and limitations, including neurovascular and multi-lineage systems. We further highlight emerging innovations that enhance the physiological fidelity, reproducibility, and scalability of these models. Collectively, these platforms establish tumor microenvironment interactions as a central organizing principle of glioma biology and provide a strong foundation for mechanistic discovery, therapeutic development, and personalized neuro-oncology.

## 1 Introduction

Gliomas comprise a diverse and highly adaptive class of primary brain tumors whose biological complexity has long challenged mechanistic understanding and therapeutic progress. Advances in molecular profiling have fundamentally reshaped glioma classification, revealing that tumors previously grouped by histology instead represent genetically and biologically distinct entities defined by specific molecular alterations and developmental context (1). In adults, glioblastoma (GBM) remains the most aggressive and lethal glioma subtype and is defined as an IDH-wild-type diffuse astrocytic glioma characterized by genetic alterations such as TERT promoter mutation, CDKN2A loss, combined chromosome 7 gain and chromosome 10 loss, and extrachromosomal amplification of oncogenes including EGFR (1). Epidemiologically, GBM accounts for approximately half of all malignant primary brain tumors in adults and carries a median survival of only 14–16 months, despite aggressive multimodal therapy (2, 3). Large-scale genomic studies of GBM demonstrate that frequent recurrent alterations in a limited set of oncogenic pathways, including alterations in receptor tyrosine kinase signaling pathways, cell cycle checkpoints, and tumor suppressor loss (4). However, these shared features obscure profound intertumoral heterogeneity, with individual tumors exhibiting distinct combinations of genetic drivers, epigenetic states, and transcriptional programs.

Beyond GBM, diffuse gliomas encompass additional molecularly defined entities. These tumors differ markedly in prognosis, treatment sensitivity, and progression trajectories, yet similarly evolve through stepwise genetic and epigenetic alterations over time (1, 5). Importantly, both high-grade and lower-grade adult gliomas, including adult IDH-mutant astrocytomas and oligodendrogliomas, are strongly shaped by interactions with their surrounding cellular microenvironment, raising fundamental questions regarding how glioma cells engage with the nervous system. Such questions are difficult to address using conventional two-dimensional tumor cell monocultures or genetically engineered animal models. Pediatric gliomas further illustrate how tumorigenesis hijacks developmental mechanisms across the lifespan. These tumors are thought to arise primarily from disruptions of neurodevelopmental and epigenetic programs rather than from the accumulation of numerous somatic mutations typical of adult gliomas (6, 7). Frequent alterations in pediatric gliomas, including diffuse midline glioma (DMG), involve histone mutations, such as H3-K27M, that globally reprogram chromatin states and differentiation trajectories, producing aggressive tumors with relatively simple genomes but profound transcriptional and epigenetic dysregulation (8–10). Multi-omics indicate that both adult and pediatric glioma tumor cells demonstrate phenotypes that are heavily influenced by interactions with surrounding neural and stromal cells types (5, 11–14), emphasizing the importance of developmentally and physiologically relevant experimental contexts.

A unifying feature among these gliomas is their extensive intratumoral cellular heterogeneity within a complex neural microenvironment. Single-cell and single-nucleus transcriptomic profiling, along with multi-omics profiling studies have shown that GBM cells occupy dynamic and plastic cellular states defined by transcriptional heterogeneity, including neural progenitor-like (NPC-like), oligodendrocyte progenitor-like (OPC-like), astrocytic-like (AC-like), mesenchymal-like (MES-like) states, as well as more recently defined subsets and refined definitions of these cellular phenotypes, which resemble early developmental subpopulations, including immature astrocytes, neuronal progenitors, and radial glia (11, 14–16). These states coexist within individual tumors and interconvert over time in response to genetic alterations, clonal evolution, therapeutic pressure, and regional microenvironmental cues (5, 11–13). In parallel, the glioma microenvironment comprises neurons, astrocytes, oligodendrocytes, vascular cells, resident microglia, infiltrating macrophages, and in some contexts, adaptive immune populations. These non-malignant cells actively shape tumor proliferation, invasion, neural circuit integration, immune evasion, and treatment response in regionally specific ways, as emphasized by integrative analyses showing that glioma progression is driven not only by tumor-intrinsic evolution but also by reciprocal tumor extrinsic microenvironment interactions (reviewed in 17).

Despite these advances, fundamental open questions remain. How do neurons, glia, and other microenvironmental components engage with glioma cells to regulate tumor growth and invasion? How does the neural microenvironment influence tumor cell plasticity, genetic instability, clonal evolution, and therapeutic response and resistance? Which aspects of tumor microenvironment crosstalk are reversible, and how are these interactions reshaped by therapy? Addressing these questions requires experimental models that preserve human tumor-specific genetics, spatial organization, lineage identity, and cell-cell interactions, particularly those governing tumor-neuron communication.

Animal models have been indispensable for uncovering core principles of glioma biology and testing therapeutic strategies *in vivo*. However, substantial differences between animals and humans in cortical organization, immune composition, and neurodevelopmental timing, limiting their ability to fully recapitulate human-specific mechanisms of glioma initiation, progression, and tumor-neuron interactions. These limitations have motivated the development of complementary human experimental systems. Early efforts centered on organotypic brain slice cultures, which preserve native architecture and enable direct tumor interrogation *ex vivo*, but are limited by restricted scalability and lifespan and model only advanced tumors (18–20), motivating the development of alternative human-specific platforms.

The advent of tissue engineering platforms, such as human induced pluripotent stem cell (iPSC) technologies (21), transformed this landscape by enabling generation of three-dimensional (3D), self-organizing organoids that recapitulate key stages of human neurodevelopment and disease processes (22). For example, iPSC-derived cerebral organoids can form ventricular- and subventricular-like zones, establish radial glial scaffolds, and generate diverse neuronal lineages providing developmentally relevant context for studying glioma-microenvironment interactions (23). Subsequent refinements including directed patterning, enhanced neuronal maturation, and incorporation of additional microenvironmental components, have further expanded the utility of these systems (24, 25). Building on these advances, a diverse array of glioma organoid platforms has emerged, including genetically engineered neoplastic cerebral organoids (NeoCOR), glioma cerebral organoid co-culture (GLICO) systems, and multiple patient-derived glioma organoid formats that retain intratumoral heterogeneity and key microenvironment features (26–30). Many of these platforms are suited for interrogating reciprocal signaling between tumor cells and the neural microenvironment, facilitating direct study of tumor-neuron interactions.

**Figure 1:**
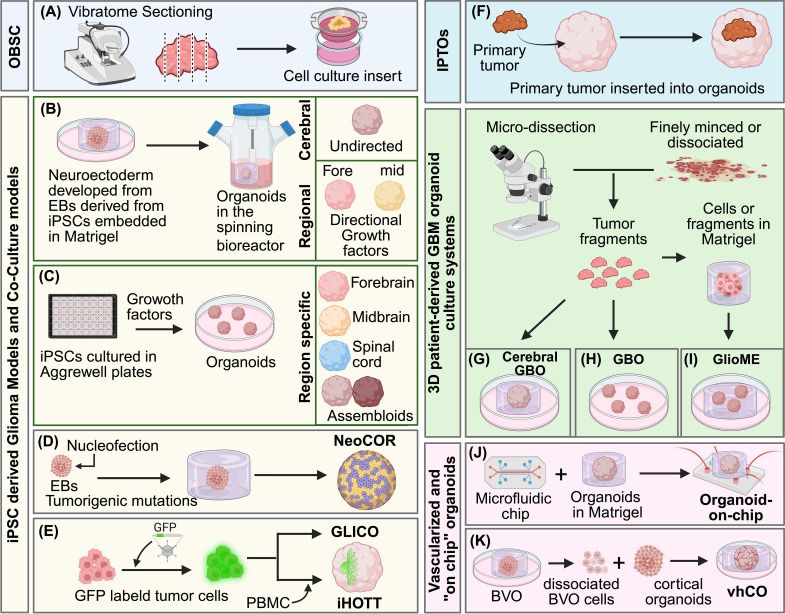
Overview of organoid-based models used to study glioma-microenvironment interactions. **(A)** Organotypic brain slice cultures (OBSCs) are generated by vibratome sectioning of resected human brain tissue and maintained on semi-permeable membranes, preserving native tissue architecture and cell-cell interactions *ex vivo*. **(B)** iPSC-derived cerebral organoids are generated through embryoid body (EB) formation and neuroectoderm specification, followed by long-term culture in spinning bioreactors. Both self-patterned and regionally specified organoids (e.g., forebrain and midbrain) can be produced using defined growth factor conditions. **(C)** Region-specific organoids and assembloids are generated from iPSCs cultured using controlled patterning in AggreWell plates and defined growth factors to model distinct neural regions (e.g., forebrain, midbrain, and spinal cord). **(D)** Neoplastic cerebral organoids (NeoCORs) are generated by introducing oncogenic mutations into iPSC-derived EBs and neural organoids, enabling modeling of glioma initiation within a developing human neural context. **(E)** Glioma–cerebral organoid co-culture (GLICO) and their derivatives are generated by engrafting GFP-labeled patient-derived glioma cells into iPSC-derived cerebral organoids. Immune human organoid tumor transplantation (iHOTT) systems further incorporate patient-matched peripheral blood mononuclear cells (PBMCs) to establish an autologous peripheral immune compartment. **(F)** Individualized patient tumor organoids (IPTOs) are generated by inserting intact primary tumor tissue into cerebral organoids, preserving tumor architecture and facilitating analysis of tumor–neural interactions in a patient-specific context. **(G–I**) Three-dimensional patient-derived GBM organoid platforms are generated through microdissection of primary tumor tissue and include cerebral organoid-based co-cultures **(G)**, glioblastoma organoids (GBOs); **(H)**, and glioma microenvironment-retaining organoids (GlioME); **(I)**, which differ in the extent to which tumor structure, cellular heterogeneity, and microenvironmental features are preserved. **(J)** Organoid-on-chip platforms integrate brain organoids embedded in extracellular matrix scaffolds (Matrigel) with microfluidic devices, allowing controlled media perfusion and modulation of microenvironmental conditions. **(K)** Vascularized human cortical organoids (vhCO) are generated by combining dissociated cells from vascularized brain organoids (BVOs) with cortical organoids, introducing vascular-like elements into neural organoid systems. BioRender used to create image graphics.

**Figure 2:**
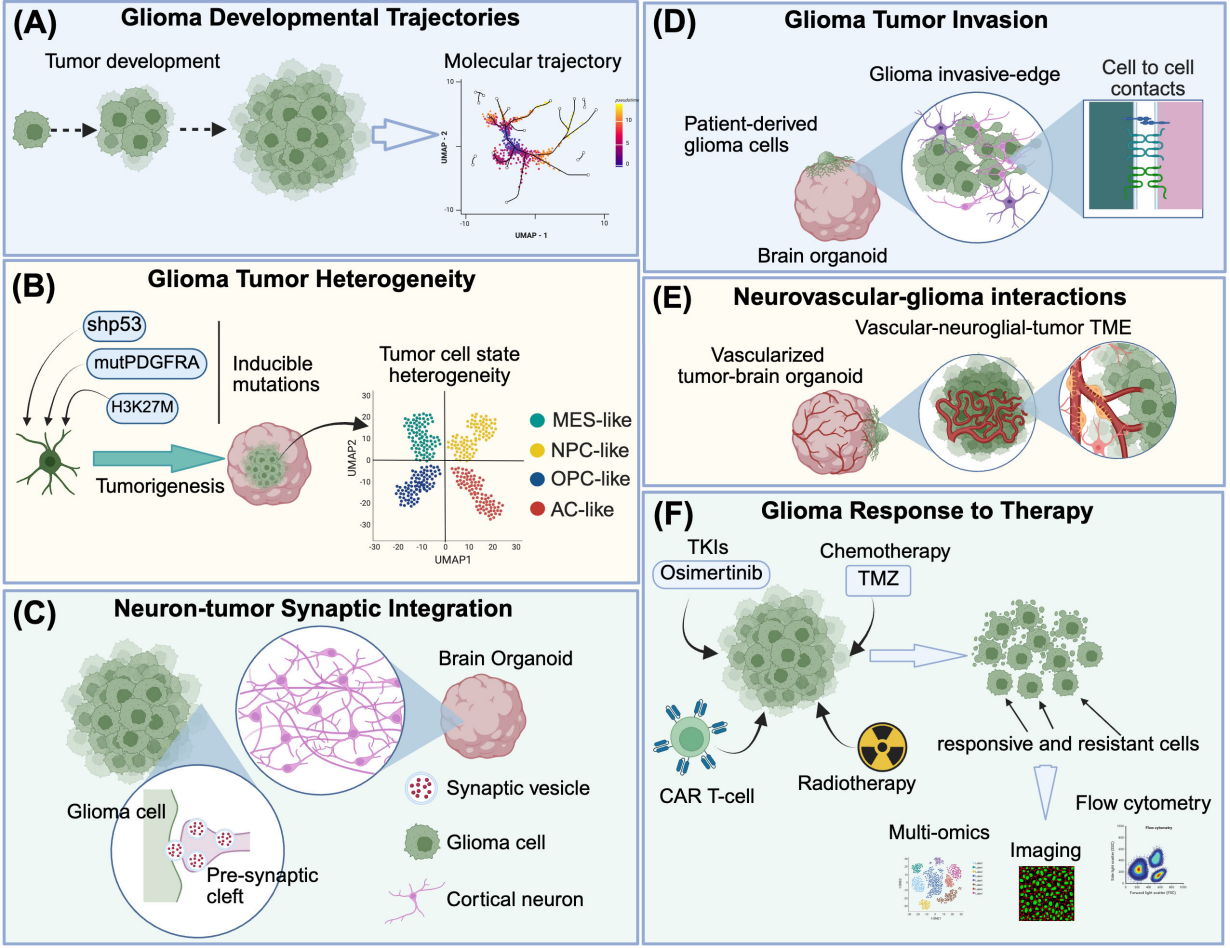
Applications of organoid models in the study of glioma biology. Brain organoids present several strengths as glioma research platforms. **(A)** Organoid models support analysis of tumor cell developmental trajectories using lineage tracing, barcoding approaches, and live imaging. **(B)** Organoid systems foster tumor cell heterogeneity, permitting molecular profiling of cell state dynamics and associated regulatory programs and phenotypic transitions. **(C)** Organoid co-culture models facilitate investigation of neuron–tumor interactions, including synaptic communication, using imaging, electrophysiology, and multi-omic approaches. **(D)** Brain organoids provide defined neural microenvironments that promote tumor invasion and influence cell state phenotypes **(E)** Integration of endothelial cells or fusion with vascular organoids introduces vascular-associated components for studying tumor–neurovascular interactions. **(F)** Organoid models can be used to assess responses to perturbations, including pharmacologic therapies, radiation, and cell therapies, and to examine mechanisms of therapeutic response and resistance among heterogeneous tumor cells in a neural microenvironment. BioRender used to create image graphics.

The major goal of this review is to survey glioma organoid model systems (**Figure 1**; **Table 1**) and synthesize their applications for studying tumor-neuron interactions, with a focus on developmental mechanisms. We will highlight innovations and adaptations that extend their capabilities (**Figure 2**), with emphasis on rigorous interpretation, benchmarking of developmental stages, and integration of complementary methodologies (**Table 2**). Moreover, we discuss their respective strengths, biological limitations, technical improvements, and current and emerging applications of these models for understanding glioma biology. Of note, because the application of glioma organoids to immunotherapy discovery and industrial drug testing has been covered in other recent reviews (55–58), we will not cover these important applications of glioma organoid models in depth. These *ex vivo* models represent a major advance toward greater physiologic fidelity among experimental models of the human glioma microenvironment. When interpreted within their experimental and developmental contexts, glioma organoid systems provide adaptable human specific platforms for investigating mechanisms underlying tumor-microenvironment interactions.

**Table 1:** Key advances in brain and glioma organoid models.

Year(s)	Model / advance	Key conceptual advance	Relevance to glioma biology	Key references
2007, 2013	Organotypic brain slice cultures	Preserves native brain architecture and cellular composition *ex vivo*	Maintains tumor cytoarchitecture and enables analysis of tumor–brain interactions in intact tissue	(18–20)
2013	Self-patterned cerebral organoids	iPSCs self-organize into 3D neural tissues with ventricular zones and radial glia	Establishes a human neurodevelopmental context with relevant neural cell types	(23, 31, 32)
2015	Directed cortical organoids (hCOs)	Morphogen-guided patterning yields reproducible cortical identity	Provides defined neuronal and glial populations for studying tumor–neuron and tumor–glia interactions	(24, 25, 33–35)
2015–2021	Region-specific brain organoids	Directed differentiation generates distinct neural territories	Enables analysis of region-dependent effects on glioma cell phenotypes	(36–42)
2016	Matrix-embedded GBM organoids	ECM-supported 3D tumor growth with hypoxia gradients	Recapitulates GSC niches, maintains GSC states, models intratumoral heterogeneity	(27)
2017	Assembloids	Fusion of region-specific organoids promotes inter-regional interactions and long range migration	Models developmental migration programs and inter-regional neuronal interactions	(36)
2018	NeoCOR (neoplastic cerebral organoids)	Genetic engineering induces focal mosaic oncogenic transformation in organoids	Models early cell-type specific gliomagenesis and links defined mutations to cellular phenotypes	(26)
2018–2019	Vascularized brain organoids	Incorporation or induction of endothelial-like cells or other vascular cell types	Introduces vascular-associated cell types to improve representation of brain microenvironment, but lacks functional perfusion	(43–46)
2018	Organoid-on-chip	Integration of organoids with microfluidic systems	Enables control of microenvironmental composition and conditions, perfusion, and mechanical forces	(47, 48)
2019	GLICO (glioma–cerebral organoid co-culture)	Engraftment of patient-derived GSCs into iPSC-derived organoids	Recapitulates invasion, tumor–neuron interactions, maintenance of tumor cell heterogeneity, and therapeutic responses	(29)
2019	GBOs (glioblastoma organoids)	Culture of intact tumor fragments without dissociation	Preserves tumor tissue structure, hypoxia gradients, and cellular heterogeneity, including innate immune cells short-term	(28)
2019	Long-term maturation / air–liquid interface organoids	Extended culture improves oxygen delivery and enhances neuronal and glial maturation and network activity	Supports investigation of tumor interactions in more mature neural environments	(49)
2021, 2026	H3-K27M-mutant DMG organoid models	Introduction of oncogenic mutations into iPSC-derived organoid systems	Enables analysis of developmental context and mechanisms of tumor initiation	(42, 50)
2025	GlioME (glioma organoids with microenvironment),	Retention of stromal and immune components in culture	Maintains aspects of tumor-associated microenvironment (early culture)	(30)
2025	IPTO (individualized patient tumor organoid)	Non-dissociated patient tumor tissue explants grafted to iPSC-derived neural organoids	Maintains stromal structure and cell types, including innate immune cells for 2–8 weeks.	(51)
2025	Pediatric HGG patient-derived organoids	Direct culture of pediatric tumor tissue	Recapitulates genetic and transcriptional features of pediatric gliomas	(52)
2026	iHOTT (immune–tumor organoid co-culture)	Co-engraftment of tumor cells with patient--matched peripheral immune cells	Enables analysis of tumor–immune interactions in a neural organoid context	(53, 54)

Models are organized chronologically to highlight key conceptual advances in the development of human neural and glioma organoid systems.

**Table 2:** Functional capabilities and limitations of glioma organoid model systems.

Model / platform	Biological context modeled	Biological concepts best addressed	Key strengths	Key limitations
Organotypic brain slice cultures	Native adult brain–tumor interface	Drug diffusion, mature ECM effects, oxygen and nutrient gradients, and age-associated microenvironmental influences	Preserves adult brain architecture and diffusion properties	Limited culture duration; low throughput
iPSC-derived organoids	Developmentally patterned human neuroepithelium	Influence of developmental cues on tumor cell state, lineage plasticity, and tumor–neuron interactions	Controlled, reproducible systems; defined neural context	Immature ECM and metabolism; lacks adult and aged microenvironment
GLICO	3D neuron–tumor interface with synaptic integration	Neuron–tumor signaling, calcium dynamics, and invasion behavior	Recapitulates neuron-dependent invasion; supports mechanistic analysis; uses both primary tumor cell cultures or dissociated tumor cells	Developmental context relative to adult tissue; hypoxia with prolonged culture; diffusion mediated drug exposures
NeoCOR	Defined oncogenic alterations in neuroepithelial scaffold	Functional effects of specific oncogenic mutations	Controlled genetic background; enables causal analysis of oncogene function	Limited to early steps of tumorigenesis; restricted tumor heterogeneity
IPTO	Patient tumor tissue integrated into neural organoid scaffold	Preservation of tumor heterogeneity with neural context; tumor-intrinsic vs microenvironmental effects	Preserves tumor architecture, heterogeneity, and stromal components, including innate immune cells (early culture)	Requires intact patient tissues; limited scalability; stromal decline over time
GBOs	Patient-derived tumor tissue with native structure	Maintenance of tumor heterogeneity and short-term microenvironmental features	Preserves tumor architecture, heterogeneity, and stromal components (early culture)	Requires patient tissues; inter-tumoral variability; hypoxia with prolonged culture; loss of stromal components over time
GlioME	Tumor–microenvironment co-culture with immune and stromal components	Influence of immune and stromal interactions on tumor cell behavior	Incorporates multiple microenvironmental components	Limited standardization; reduced scalability
H3-K27M-mutant DMG organoid models	Pediatric glioma development in neural context	Cell of origin and developmental drivers of tumorigenesis	Enables analysis of developmental tumor initiation	Limited availability of patient material; lacks mature ECM and vasculature
iHOTT	Tumor cells with patient-matched peripheral immune cells in organoid context	Short-term tumor–immune interactions	Enables controlled analysis of tumor–immune interactions	Limited immune diversity; developmental neural context
Vascularized brain organoids	Neural organoids with endothelial-like cells	Tumor–neurovascular interactions and vascular-associated migration	Introduces vascular cell types	Lacks functional perfusion and BBB properties
Organoid-on-chip	Microfluidic organoid systems with controlled flow	Effects of perfusion, shear stress, and microenvironmental dynamics	Enables controlled physical and chemical conditions	Technically complex; lacks fully functional BBB without additional components

Models are grouped by biological context and experimental capabilities. ECM, extracellular matrix; GSC, glioma stem cell; BBB, blood–brain barrier.

## 2 Organoid and assembloid models to study glioma microenvironment interactions

A range of organoid-based platforms has been developed to model glioma biology within human neural tissue contexts. In this section, we outline the major classes of these models, highlighting their experimental design, biological relevance, and key strengths and limitations for studying tumor microenvironment interactions.

### 2.1 Human organotypic brain slice culture

Organotypic brain slice cultures represent the earliest tissue-based platforms for modeling glioma within an intact human brain microenvironment (59, 60), and are often regarded as prototypical organoid-like systems in neuro-oncology (**Figure 1A**). By preserving native tissue architecture, cellular composition, and local cell-cell interactions *ex vivo*, these models enable direct interrogation of tumor biology within human neural tissue and continue to serve as an important benchmark for evaluating newer 3D models. Organotypic slice cultures generated from freshly resected GBM tissue retain key histopathological features of parent tumors, including pseudopalisading architecture, nuclei atypia, neovascularization, and necrotic foci (18–20). Following vibratome sectioning and placement on semi-permeable membranes, these preparations remain viable for several weeks, permitting functional assessment of responses to chemotherapy and radiotherapy. Although tissue integrity can vary among samples, methodological refinements, such as use of tissue choppers in place of vibratome sectioning, have improved reproducibility and tissue survival (18). Complementing tumor-derived slice cultures, Ravi and colleagues developed methods for maintaining surgically resected non-tumor human cortical tissue in long-term culture without significant loss of viability (19). Microinjection of patient-derived GBM cells into these slices reconstructed tumor growth within a human neural environment, yielding invasive tumors cells, reactive astrocytes in the host slice, and treatment responses resembling those observed *in vivo* (19).

Despite their strengths, organotypic slice culture approaches have inherent limitations. Reliance on surgically resected tissue limits their use for modeling early tumor initiation, and genetic manipulation of tumor or microenvironmental populations remains technically challenging. In addition, slice cultures capture only localized regions of tumor-infiltrated brain tissue, limiting analyses of longer-range invasion. Practical constraints, including limited access to human brain tissue and variability in tissue quality and longevity, further constrain their use, underscoring the need for complementary human-based model systems with greater scalability and experimental flexibility (**Table 2**).

### 2.2 iPSC-derived glioma organoid and co-culture models

In this section, we describe how advances in human induced pluripotent stem cell (iPSC) technology have been adapted to engineer glioma organoids and their derivatives, including co-culture and assembloid platforms, for investigating tumor initiation, heterogeneity, and interactions with the neural microenvironment.

#### 2.2.1 iPSC-derived brain organoids

Human iPSC technology has enabled researchers to move beyond limitations inherent to earlier human tissue-based glioma model systems by providing access to developmentally defined human neural tissues *in vitro* (21). Early self-patterned cerebral organoids formed multiple neural regions and developed functional neuronal networks exhibiting spontaneous activity (23, 31, 32). Embedding iPSC-derived embryoid bodies in Matrigel and culturing them in spinning bioreactors, which enhance oxygen and nutrient diffusion, permits sustained growth and intrinsic tissue patterning into self-assembled cerebral organoids that recapitulate key features of the developing human brain, including ventricular- and subventricular-like regions populated by radial glia and neurons (23) (**Figure 1B**). However, these unguided organoids exhibit considerable variability in size, structural morphology, regional identity, and cell type composition. To address this variability, Velasco et al. (33) compared self-patterned (23, 31, 32) and directed forebrain organoids (34, 35), finding that dorsally patterned cortical organoids generated using defined morphogen cues reproducibly yielded a consistent repertoire of cortical cell types that followed developmental trajectories similar to those of the developing human cortex. Moreover, organoids derived from distinct iPSC lines show highly comparable transcriptional profiles, indicating that directed protocols using defined conditions and validated cell lines substantially reduce inter-organoid variability while preserving human cortical diversity (33).

Paşca and colleagues further advanced iPSC-based approaches by developing directed, forebrain-specific human cortical organoids (hCOs) (24) (**Figure 1C**) without the use of Matrigel or bioreactors. The resulting hCOs recapitulate ventricular and subventricular zones, radial glial scaffolds, neuronal migration, and progressive maturation of cortical neurons and astrocytes (24, 61, 62). Sloan et al. demonstrated that long-term organoid culture yields mature human astrocytes (62). Trujillo et al. (25) documented functional maturation of neurons in cortical organoids, which exhibited electroencephalographic patterns reflecting spontaneous network activity and coordinated electrical signaling within developing neural circuits. Together, these results established that hCO models reproduce early neurogenesis and subsequent gliogenesis.

Subsequent refinements have improved the fidelity of cortical organoid models. Standardized protocols now yield reproducible forebrain organoids, with consistent regional forebrain identity across multiple iPSC lines and defined benchmarks for organoid quality control (63). Air-liquid interface methods promote surface oxygenation and nutrient access, further enhancing neuronal survival, long-range synaptic connectivity, and extended culture viability (49). Long-term human brain organoid cultures can exhibit extensive cell-type diversity and functional neuronal networks (32), underscoring their capacity to model both structural organization and functional maturation of the human brain. Despite these advances, the absence of vascular networks remains a key limitation, restricting oxygen and nutrient diffusion and limiting stromal composition, leading to necrosis in larger organoids subject to prolonged culture, thereby indicating the need for alternative strategies to sustain long-term maturation and enhance stromal complexity (**Table 2**).

#### 2.2.2 Genetically engineered glioma organoid models

The use of cerebral organoids to model gliomagenesis has been refined through controlled genetic engineering strategies that directly link defined oncogenic alterations to emergent tumor phenotypes in 3D human neural tissue models. For example, neoplastic cerebral organoids (NeoCORs) represent a key advance in modeling tumor initiation within a physiologically relevant human context (26) (**Figure 1D**). Bian and colleagues established the NeoCOR platform using Sleeping Beauty (SB) transposon-mediated oncogene insertion to generate focal, mosaic neoplastic transformation within otherwise normal cerebral organoids (26). NeoCORs recapitulate hallmark features of high-grade glioma, including expansion of progenitor-like tumor cell populations and associated cytoarchitectural disruption. Upon transplantation into immunodeficient mice, NeoCORs show invasive behavior and phenotypes consistent with epithelial-to-mesenchymal transition. This system permits systematic evaluation of how individual mutations influence proliferation, invasion, and therapeutic sensitivity during early gliomagenesis. For example, pharmacologic inhibition of EGFR signaling selectively suppresses tumor cell growth in EGFR-mutant NeoCORs, demonstrating preserved driver-dependent activity within a human brain organoid (26). These features establish NeoCORs as a mechanistically tractable platform for interrogating mutation-specific dependencies within a developmentally organized neural environment (**Table 2**).

Complementary tissue engineering approaches have further expanded these approaches. For example, using human embryonic stem cell-derived cerebral organoids, Ogawa et al. (64) established a genetically engineered GBM model through CRISPR/Cas9-mediated disruption of TP53 in defined cell subsets. This manipulation induced focal malignant transformation characterized by increased proliferation and formation of tumor-like masses within the organoid parenchyma. Malignant cells exhibited infiltrative expansion into adjacent areas and, and when transplanted into naïve cerebral organoids, engrafted efficiently and reproduced neoplastic phenotypes (64), recapitulating pathological features of human GBM. Collectively, these studies support the utility of engineered cerebral organoids as experimentally tractable models for investigating genetic and cellular origins of glioma.

#### 2.2.3 Glioma stem cell–organoid co-culture models

Understanding mechanisms of sustained GBM progression and therapeutic resistance requires experimental systems that faithfully represent the cellular complexities of these tumors. Neurosphere cultures of patient-derived glioma stem cells (GSCs), maintained in defined serum-free media supplemented with growth factors, present physiologically relevant experimental models that preserve neural stem cell-like features observed in tumor cells in the brain (65, 66). However, prolonged neurosphere culture promotes clonal selection and adaptation to culture conditions, resulting in genetic and phenotypic drift away from native tumor cell states, limiting the ability of these cultures to recapitulate tumor biology.

The GLICO model addresses these limitations by grafting patient-derived GSCs into iPSC-derived cerebral organoids (29) (**Figure 1E**). Following engraftment, GSCs invade host organoids and form tumors that recapitulate key GBM histopathological features. Distinct patient-derived GSCs retain their intrinsic mutations and invasive behaviors in this system, preserving clinically relevant heterogeneity that is often lost in conventional culture. Notably, EGFR amplification, frequently present as extrachromosomal amplicons in GBM (67, 68), is reduced or lost during extended GSC monoculture but remains preserved within the organoid microenvironment (29).

Comparative studies demonstrate that GSC proliferation within GLICOs depends on signals from the neural niche, highlighting the role of the microenvironment in sustaining tumor growth (69, 70). GLICOs also develop complex tumor microtubules (TM) networks reminiscent of those observed in GBM, including multicellular projections that support calcium signal propagation, coordinated invasion, and increased stress resistance in response to therapeutics (71). Therapeutic responses differ substantially between cultures conditions, and, while GSCs in neurospheres exhibit marked cytotoxicity in response to chemotherapies or ionizing radiation, GSCs in GLICOs demonstrate enhanced resistance that more closely parallels clinical responses in GBM (29). Thus, GLICO models can faithfully reproduce the molecular and functional complexities of patient tumors, establishing them as powerful platforms for preclinical research on GBM biology and therapy (**Table 2**).

Derivatives of the GLICO approach have been used to investigate the properties of GBM cell populations directly isolated from patient tumors, which can be experimentally manipulated following engraftment into iPSC-derived cortical organoids. These systems have been used to analyze intrinsic de-differentiated tumor cell states and to identify extrinsic cues from the neural microenvironment that promote these states. For example, Bhaduri et al. observed that tumors are enriched for a distinct subpopulation of proliferative cells also present in primary tumors that transcriptionally, phenotypically, and morphologically resemble outer radial glia (ORG) (14), a subset of radial glial stem/progenitor cells normally confined to embryonic cortical development (72). In organoids, ORG-like GBM cells exhibited asymmetric mitotic behavior associated with self-renewal that generate highly migratory daughter cells that extensively infiltrate the organoid parenchyma (14), highlighting the utility of organoid systems for investigating how progenitor-like tumor cell subpopulations contribute to tumor growth and invasion.

In a subsequent study, Ge et al. used GLICO-like systems to identify and interrogate receptor-ligand interactions between patient-derived tumor cells and the neural microenvironment (**Figure 1E**) (53). These analyses demonstrated that select stromal components from parental tumors are retained within organoid co-cultures, although immune cell populations remain limited and other stromal cell types, including differentiated oligodendroglia and endothelial cells, are not maintained. In this context, PTPRZ1, a receptor tyrosine phosphatase primarily expressed by neurons within the cortical organoid niche, was shown to promote radial glial-like tumor cells states and restrict more aggressive MES-like states and vascular cell recruitment, likely through midkine family ligand-receptor interactions (53). PTPRZ1 also regulates radial glial cell migration during early development, suggesting that its role in tumor–neuron interactions may reflect conserved aspects of its normal function in radial glial cell development (14).

Together, these studies demonstrate that GLICO-type models can be extended to capture tumor heterogeneity and define microenvironmental signals that shape tumor cell state and tumor-neuron interactions. However, they also indicate that these models offer limited representation of stromal compartments and cell types. Emerging techniques designed to improve the incorporation of stromal elements, such as vascular components to improve these models are discussed below.

#### 2.2.4 Region-specific brain organoids and assembloids

Generation of region-specific brain organoids has further advanced modeling approaches for human neurodevelopment (**Figure 1C**). Complementary protocols have been established to generate ventral forebrain (subpallial) cortical organoids with interneurons (36). These protocols, typically involve inhibition of WNT signaling and activation SHH signaling, combined with allopregnanolone to stimulate neuronal maturation and retinoic acid (RA) to refine ventral identity, followed by treatment with BDNF and NT3 to promote neuronal differentiation and synaptic maturation, yielding GABAergic interneurons (36).

A major advance arising from these studies is the generation of forebrain assembloids, created by fusing region-specific organoids to model long-range neural interactions. When dorsal and ventral forebrain organoids are positioned adjacent to one another, they spontaneously fuse and establish a functional interface through which interneurons migrate from ventral to dorsal compartments and integrate into neural circuits, including excitatory circuits between ventral GABAergic interneurons and cortical glutamatergic neurons (36). This assembloid platform enables real-time imaging and pharmacological manipulation of interneuron migration and circuit assembly, facilitating investigation of how glioma cells exploit developmental programs to infiltrate and integrate into human neural tissue.

Beyond the forebrain, region-specific differentiation strategies have been developed to model additional brain territories. Midbrain organoids can be efficiently generated from human iPSCs directed by midbrain-patterning morphogens, including SHH and FGF8, leading to formation of ventricular-like zones and dopaminergic neuron populations (37, 38), which can also be generated using simplified static culture systems (39). Similarly, region-specific protocols have been devised to generate hindbrain organoids to model embryonic caudal neural regions. For example, Muguruma et al. (40) demonstrated that human iPSCs can self-organize into polarized cerebellar tissue under defined 3D conditions driven by FGF19 and SDF1, producing cytoarchitecture reminiscent of the embryonic cerebellum (40). Additional region-specific models include iPSC-derived cervical spinal cord organoids as well as brainstem organoids enriched for pontine and medulla specific neuronal and glial lineages (41, 42).

These approaches have established model systems for studying regionally specialized neural differentiation and disease mechanisms for the midbrain, hindbrain, brain stem, and spinal cord. These models recapitulate region-specific neuronal and glial populations absent from conventional neurosphere culture or animal model systems and facilitate direct investigation of interactions between heterogenous glioma cells with regionally defined neural cell populations and neural circuitry, as illustrated in examples discussed in Section 3 (42, 73).

### 2.3 Tissue explant–based glioma organoid models

iPSC-based GBM organoid models, including GLICO and related systems, are limited by the loss of native tumor tissue structure during dissociation and purification of tumor cells prior to engraftment. To address these limitations, the individualized patient tumor organoid (IPTO) model was recently developed by engrafting non-dissociated patient tumor tissue explants onto iPSC-derived cortical organoids (**Figure 1F**) (51). IPTOs have been generated from both GBM and lower-grade gliomas, with tumor cells observed infiltrating throughout host cortical organoids.

In GBM-derived IPTOs, tumor cells display high proliferative activity and recapitulate diverse NPC-like, AC-like, and MES-like GBM transcriptional states. Notably, IPTOs retain key non-neoplastic stromal populations, including microglia, tumor-associated macrophages, T-cells, and vascular components. These stromal elements organize into perivascular niches and tumor-associated microvasculature-like structures, reflecting stromal features of the parent tumors. Comparative histopathological and molecular analyses demonstrate close concordance between IPTOs and their matched parent tumors. Innate immune populations are particularly well represented during early culture, especially within the first 2 weeks following engraftment, with histologic evidence supporting persistence of myeloid-lineage cells for up to 8 weeks (51).

By preserving heterogeneous tumor and stromal populations within a structured neural context, IPTOs enable investigation of tumor-neural dynamics, including invasion patterns and lineage plasticity, in the presence of stromal elements. These features position IPTOs as representative patient-specific preclinical models and support their broader application, given their improved preservation of stromal complexity relative to GLICO systems (**Table 2**).

### 2.4 Patient-derived three-dimensional GBM organoid systems

Despite advances enabled by iPSC-derived brain organoid platforms, these models typically rely on dissociated tumor cells and tissue explants, limiting preservation of endogenous spatial architecture, especially of more hypoxic tumor core regions, and they incompletely support the diverse stromal cell populations and structures that define the glioma tumor microenvironment. To address these limitations, multiple approaches have been developed to generate *ex vivo* organoids directly from resected patient tumor tissues, with the goal of maintaining molecular, cellular, and tissue-level features of gliomas.

Hubert et al. (27) developed a method to generate high-fidelity GBM organoids directly from patient tumor tissues (**Figure 1G**). In this approach, tumor specimens are embedded and cultured on orbital shakers, promoting formation of organoids that preserve structural organization and tumor heterogeneity. A defining feature of these organoids is the emergence of intrinsic oxygen and nutrient gradients that drive regional specialization of GSC niches as they mature. Cells at the periphery proliferate more rapidly and exhibit increased apoptosis, whereas hypoxia-adapted GSCs within the core regions divide more slowly and display enhanced survival and radio-resistance, consistent with established GSC phenotypes (27). These observations indicate coexistence of multiple GSC states within a single organoid. Heterogeneity among GSC populations is further reflected by regional variation in expression of stemness-associated regulators, such as TLX (74, 75) and OLIG2 (76–78), which show variable co-expression with SOX2 across proliferative and hypoxic compartments. Importantly, organoids generated from spatially distinct tumor regions retain functional differences reflective of their origins. For example, organoids derived from invasive tumor margins and necrotic tumor cores show distinct growth dynamics, with GSCs derived from necrotic cores displaying enhanced tumor-initiating capacity upon xenograft (27). Overall, this method generates GBM organoids that preserve structural organization, regional specialization, and intratumoral heterogeneity, supporting its utility for mechanistic and translational studies (79).

In a second complementary approach, GBM organoids (GBOs) are generated directly from fresh surgical tissue without enzymatic or mechanical dissociation (28) (**Figure 1H**). In this method, tumor specimens are trimmed and sectioned into small (~1 mm) pieces of intact tissue and cultured on orbital shakers, where they reproducibly form rounded organoids within 1–2 weeks. These organoids can be propagated through mechanical sectioning while maintaining native tissue microstructure and stromal cell components. Notably, innate immune cell populations are detectable early in culture, persist for 2–3 weeks, and exhibit transcriptional profiles consistent with those of the parent tumors (28). Histopathological and molecular analyses demonstrate that GBOs recapitulate key tumor features, including hypoxic gradients, nuclear atypia, infiltrative growth, and diverse GSC-like and neuro-glial progenitor-like subpopulations during *ex vivo* culture (28). Following orthotopic transplantation into immunodeficient mice, GBOs engraft efficiently, generating invasive tumors that retain genetic alterations and mirror growth kinetics and histopathology of their parent tumors (28).

A third tissue-preserving strategy, termed glioma organoids with a microenvironment (GlioME), was introduced by Zheng et al. to further maintain cellular diversity and structural organization (30) (**Figure 1I**). In this method, freshly resected glioma tissue is mechanically minced, embedded in Matrigel, and cultured under conditions that support organoid formation. Similar to GBOs, GlioME organoids retain proliferative activity, progenitor identities, histopathological structure, and intratumoral genetic and transcriptional heterogeneity. Genomic and epigenomic analyses reveal strong concordance with matched parent tumors, with conservation of driver mutations, copy number variation (CNV) patterns, transcriptional programs, and DNA methylation states. Notably, GlioMEs also retain tumor-resident immune populations that resemble those of the parent tumors, although these populations are evident at early time points in the first few weeks of culture (30). While immune cells can be detected during initial culture, conditions that promote their persistence and functional competence during extended culture remain less well defined, and await further study.

These tissue-preserving GBM organoid approaches maintain key features of primary tumors, including regional specialization, and intratumoral heterogeneity, while enabling analysis of distinct tumor cell subpopulations within humanized context. A major strength of these models is their ability to capture patient-specific tumor characteristics, supporting translational applications such as pharmacologic testing, although they are less suited for controlled genetic manipulation (**Table 2**). Important considerations include the extent to which endogenous stromal components, including vascular structures and immune populations, are maintained, functionally integrated, and sustained over time, as these elements play critical roles in tumor progression and therapeutic response. Moreover, the emergence of hypoxia and hypoxia-adapted compartments, can influence tumor cell states and significantly limit certain applications of these models, as discussed below.

### 2.5 Developmental state and microenvironmental fidelity in glioma organoid models

As organoid-based platforms are increasingly used to model the glioma microenvironment, one major challenge is accurately interpreting both the developmental stages of iPSC-derived brain organoids and the phenotypic states of tumor cells (**Figures 2A, B**). Cerebral organoids reproducibly model transcriptional and epigenomic programs of the human embryonic neocortex (80) and recapitulate key regulatory features. Multi-omic profiling demonstrates that organoids reproduce stage-specific enhancer landscapes, lineage-defining transcription factor networks characteristic of corticogenesis, and developmentally patterned DNA methylation (81–83), consistent with embryonic to neonatal states rather than mature adult states. Similarly, GBM reactivates embryonic transcriptional and epigenetic regulatory networks (11, 14, 73, 84), with tumor cells adopting neurodevelopmental programs predominantly used to define their cellular heterogeneity and functional plasticity. Comparative multi-omic and developmental trajectory analyses show that GBM cells diverge from adult lineage commitment checkpoints, suggesting that malignancy reflects persistence in and/or reversion to immature developmental states rather than completion of adult differentiation programs (14, 85). These observations indicate that GBM co-opts embryonic programs also present within cerebral organoid systems, and that these models provide a permissive niche that reinforces such states.

Neural organoids are not static and mimic aspects of human developmental progression over time. Prolonged culture promotes cellular maturation, yielding more mature astrocytes and neurons (62, 86), along with refinement of neural electrophysiological properties and increased cellular diversity, ultimately supporting neural circuit formation (32). These processes generate a spectrum of maturation states and cell types that influence tumor–microenvironment interactions. When GBM cells are maintained in more mature and cellularly diverse neural organoids, malignant states are preserved and tumor cell states that engage in tumor-neuron communication are enriched. For example, recent work by Bhatia et al. (73) using region-specific iPSC-derived brain organoids demonstrates that the neural microenvironment actively reshapes GBM cellular heterogeneity. Across forebrain, midbrain, and spinal cord organoids, GBM cells exhibit consistent shifts toward NPC-like states, defined by expression of synaptic genes and embryonic neuronal development programs, and reduction in AC-like states (73). Notably, these NPC-like states are enriched even among cells derived from tumors and primary cultures that lacked such populations, indicating that this phenotype is actively induced within the organoid niche. This effect is observed across regional organoid types, with forebrain organoids supporting greater invasion driven by interactions with excitatory neurons (see section 3.1). Mechanistically, these immature epigenetic and transcriptional programs provide more than descriptive similarity, and instead reflect dynamic tumor cell states that support lineage plasticity, proliferative capacity, and developmentally encoded migratory phenotypes (53, 87). These immature regulatory states may also contribute to therapeutic resistance, as differentiation-based therapies may not fully target tumor cells operating within embryonic-like programs (14, 85).

GBM is thought to exploit developmental regulatory logic as a driver of disease, and organoid platforms are well suited to investigate these processes. However, important questions remain regarding how faithfully these systems recapitulate the tumor microenvironment. iPSC-based organoid systems reproduce certain aspects of the glioma niche, including tumor-neuron signaling, tumor-astrocyte interactions, and spatially structured growth within a human neural tissue context (14, 29, 85, 88, 89). Patient-derived systems such as GBOs also recapitulate spatial features, including hypoxic regions (27, 28). As organoids increase in size, diffusion constraints generate hypoxic cores and metabolic zonation (90). Although hypoxia and necrosis are hallmark features of GBM *in vivo* (91), in organoids these gradients can ectopically arise from physical diffusion constraints rather than tumor-driven remodeling, complicating interpretation of whether organoid models faithfully represent these features. Hypoxia can shift tumor cells toward MES-like or stress-adaptive transcriptional programs (92), influencing cell-state distributions, while diffusion barriers can confound pharmacologic studies by limiting drug penetration (93). Additional considerations include the stability of tumor composition during prolonged 3D culture. Under size-controlled conditions, GBOs and IPTOs can be propagated for several months while retaining genetic subclones and CNV patterns present in parental tumors (28, 51). However, most protocols typically limit organoid size through repeated mechanical sectioning and restrict culture duration to minimize necrosis. However, patient-derived organoids retain regional stromal cell types short-term for only ~2–8 weeks (51). Across organoid models, continued growth alters tissue architecture, cellular composition, and diffusion gradients, and prolonged culture or passaging may introduce selective pressures that reshape clonal composition or restrict nutrient availability, leading ectopic necrosis and attenuation of intratumoral heterogeneity over time.

Experimental design and data interpretation should therefore account for diffusion-driven processes and be benchmarked by rigorous quality control, comparative metrics, and complementary approaches. For example, multi-omic data from organoid models can be credentialed against matched parent tumors, pharmacologic findings can be verified with genetic approaches, and results can be compared with *in vivo* systems that capture cellular complexities, such as intercellular interactions and tissue vascularization. To address intrinsic limitations of organoid systems, new platforms are incorporating perfusion and control of physical parameters such as organoid size and geometry (**Table 1**), including microfluidic organoid-on-a-chip technologies (**Figure 1J**) (47). These advances aim to improve physiological fidelity while preserving experimental accessibility of organoid systems.

## 3 Brain organoids as platforms to study tumor progression in the neural microenvironment

Brain organoid models provide a framework to examine tumor-neuron interactions in GBM within a humanized neural microenvironment. In this section, we examine how these systems enable dissection of synaptic, paracrine, and circuit-level mechanisms that shape tumor growth, invasion, and cellular state dynamics, and highlight how these processes potential reveal therapeutic vulnerabilities.

### 3.1 Brain organoids for studying tumor–neuron interactions

Tumor-neuron interactions have emerged as a fundamental organizing feature of GBM biology, influencing tumor growth, invasion, cellular state, and clinical outcome (**Figure 2C**). Rather than acting as passive bystanders, neurons actively shape glioma progression through activity-dependent paracrine signaling and direct synaptic communication. Brain organoid-based models have been instrumental in understanding these interactions by restoring key elements of the human neural microenvironment, including synaptically active neurons, defined neuronal subtypes, and neurophysiological signaling programs, that are absent from conventional tumor monocultures. By enabling controlled manipulation of neuronal identity, developmental state, and regional context, organoid systems provide tractable platforms for dissecting how GBM cells integrate into and adapt to neural environments to support tumor growth, propagation, and therapeutic response.

Foundational research from Monje, Venkatesh, and colleagues established that neuronal activity promotes glioma proliferation through activity-dependent secretion of paracrine factors, most notably a cleaved form of the postsynaptic adhesion protein neuroligin-3 (NLGN3) (reviewed in 94). NLGN3 release from active neurons stimulates tumor growth, induces synapse-associated gene expression programs in glioma cells (95, 96), and stimulates tumor progression through malignant neuron-to-glioma synaptogenesis (97). These observations contributed to a major conceptual advance: the recognition that GBM cells form functional synapses with neurons. Subsequent studies demonstrated that glioma cells receive excitatory glutamatergic synaptic input, undergo depolarization, and electrically integrate into neural circuits, with synaptic activity directly driving tumor proliferation (reviewed in 17).

Suppression of NLGN3 release, either genetically or pharmacologically, markedly attenuates tumor progression, establishing neuronal activity as an instructive oncogenic signal rather than a permissive feature of the microenvironment (96, 97). At the molecular level, malignant neuron–glioma synapses recruit synaptogenic and neurotrophic pathways associated with neuronal plasticity, including calcium-dependent transcriptional programs and BDNF-TrkB signaling, reinforcing connectivity and tumor growth (94, 97, 98). At the network level, tumor-neuron interactions have been linked to clinical outcomes. For example, GBM remodeling of neural circuit activity correlates with cognitive dysfunction and reduced patient survival, suggesting that integration into neural networks confers a selective advantage to tumor cells (99).

Brain organoids provide neuroglial microenvironments in which these interactions can be examined. Human cerebral organoid co-culture and invasion assays demonstrate that glioma infiltration and growth are enhanced by neuron-derived paracrine and synaptic cues, including NLGN3, confirming that organoids recapitulate activity-dependent trophic and synaptic signaling in a human context (69, 99–102). These studies validate organoid platforms as physiologically relevant systems bridging *in vitro* assays and *in vivo* models.

Building on this foundation, Sun et al. developed a hybrid connectome-scale framework integrating patient-derived GBM cells and human cerebral organoids with *in vivo* circuit mapping in the adult mouse brain, facilitating brain-wide analyses of tumor-neuron connectivity benchmarked against *in vivo* data (88, 103). Using monosynaptic rabies virus tracing, tissue clearing, and light-sheet microscopy, they demonstrated that tumors integrate into distributed neural networks spanning both local and long-range brain regions rather than forming isolated contacts. Transcriptomic profiling of synaptically connected tumor cells revealed enrichment of programs associated with synaptic signaling, calcium flux, membrane excitability, and neuronal communication, particularly among NPC-like tumor cells. Tumor cells also received cholinergic inputs from neurons through the muscarinic acetylcholine receptor 3 (CHRM3), activating pathways linked to cytoskeletal remodeling, axon guidance, and migration, implicating neuronal circuits in invasive and plastic tumor phenotypes (88, 103, 104).

Complementing this systems-level approach, Sun et al. developed a fully human tumor and iPSC-derived neuronal co-culture model to directly interrogate causal relationships among neurotransmitter-specific tumor–neuron synapses (88, 89). Using monosynaptic rabies tracing, synaptic marker analyses, and calcium imaging, the authors demonstrated functional cholinergic synapses from neurons onto tumor cells, marked by ChAT^+^/VAChT^+^ presynaptic puncta. Cholinergic signaling triggered acetylcholine-dependent calcium signaling, stimulating tumor proliferation and inducing transcriptional shifts toward NPC-like states through CHRM3. Together, these complementary approaches define both the extent and mechanistic consequences of tumor-neuron integration, and underscore the utility of organoid platforms for linking circuit topology and neurotransmitter-specific signaling mechanisms (88, 89).

Region-specific neural organoid microenvironments further reveal how developmental context shapes GBM cell behavior (73). Immature, neurogenic forebrain organoids preferentially activate cholesterol and steroid biosynthesis pathways, whereas more mature, gliogenic organoids induce VEGF- and IGF-associated signaling. Tumor cells also exhibit enrichment of synaptic and invasion-related programs, including nicotinic cholinergic, GABAergic, and glutamatergic receptors. Rabies tracing demonstrated bidirectional synaptic communication between GBM cells and host organoid neurons, with TBR1^+^/vGLUT1^+^ deep-layer excitatory neurons serving as dominant partners in forebrain models. Tumor cells connected to these neurons exhibited increased migration, linking neuron subtype-specific synaptic connectivity to invasion (73).

Emerging conceptual syntheses place GBM within a growing class of malignancies that exploit neuronal activity and synaptic communication to support growth, invasion, and therapy resistance. Across cancer types arising in or metastasizing to the brain, as well as cancers interfacing with peripheral nerves, neuron-derived signals are increasingly recognized as conserved components of tumor microenvironments (105–110), with GBM representing a prominent example of circuit-level tumor integration (111). Brain organoid models occupy a unique niche in this emerging field of cancer neuroscience, permitting controlled, human-specific investigation of neuron-driven tumor biology, while retaining key aspects of neural organization and activity.

These studies establish tumor-neuron interactions as a core feature of GBM pathophysiology that can be effectively interrogated using organoid platforms. By integrating into neural circuits and exploiting synaptic and neuromodulatory signaling, GBM cells leverage the neural microenvironment to drive disease progression. As organoid platforms evolve to incorporate additional microenvironmental components, including neuroglia, vasculature, and immune cells, they are poised to provide an increasingly comprehensive framework for identifying therapeutic strategies that disrupt these interactions.

### 3.2 Brain organoids for studying glioma invasion in neural tissue

Diffuse infiltration of glioma cells throughout the brain parenchyma is a defining and lethal feature of these tumors (**Figure 2D**). Brain organoid-glioma co-culture models, including GLICO-type systems, provide experimentally tractable platforms for studying invasion within a human neural microenvironment. These models facilitate interrogation of tumor-intrinsic programs and extrinsic cues derived from neural tissue, while permitting genetic and pharmacologic manipulation of tumor and stromal compartments to explore causal mechanisms of invasion (112). Importantly, they also enable investigation of aspects of glioma invasion that have not previously been feasible to study *in vitro*.

Proof-of-concept studies demonstrated the feasibility of organoid-based invasion assays using murine embryonic stem cell-derived brain organoids co-cultured with GBM spheroids (113). Quantitative imaging revealed preferential migration of GBM cells toward organoid cores, accompanied by increased expression of invasion-associated genes such as Vimentin and matrix metalloproteinase-9 (MMP9), relative to non-neoplastic neural progenitors. These studies established organoids as suitable substrates for analyzing glioma invasion dynamics. Subsequent research linked invasive behavior directly to tumor–neuron interactions.

Invasion dynamics have also been characterized using GLICO-like models. For example, Krieger et al. co-cultured patient-derived GBM cells with human iPSC-derived hCOs and visualized invasion using 3D confocal imaging, and observed tumor subpopulations enriched for ligands and receptors associated with ECM interactions and invasion pathways (114). Invasive GBM cells preferentially receive neuronal input while remaining weakly connected to intra-tumoral networks, suggesting that neuronal connectivity selectively supports migratory tumor states (100, 102). Venkataramani et al. further demonstrated that GBM co-opts neuronal and developmental programs associated with axonal growth, synaptogenesis, and activity-dependent migration to facilitate diffuse invasion (102). Highly invasive tumor cells decouple from tumor networks while maintaining synaptic engagement with neurons, and adopting neuronal NPC-like transcriptional features linked to migration and circuit integration (100, 102). Regionally patterned organoids further reveal how neuronal subtype and regional identity influence migration. For example, Fan et al. (115) placed patient-derived GBOs adjacent to dorsal or ventral forebrain organoids within 3D microfluidic systems, where GBM cells preferentially migrated towards dorsal forebrain organoids, exhibiting enhanced axon-guided migration and synapse formation, compared to ventral organoids. These interactions are accompanied by ECM remodeling and bidirectional electrophysiological signaling between tumor and neural cells, reinforcing invasion and growth.

GLICO-like systems also recapitulate multicellular TM networks that enable long-range tumor interconnectivity, migration, and therapeutic resistance (29, 71). TM formation correlates with invasion depth, although they vary by tumor of origin (116). Bioinformatic analyses of ligand-receptor interactions reveal reciprocal signaling, with tumor cells inducing neuronal programs and host organoid cells providing ECM cues that facilitate invasion. Similarly, GBM explant-organoid assembloids (GCOAs) facilitate longitudinal analyses of invasion. Within GCOAs, tumor cells exhibit amoeboid, mesenchymal, and collective migration modes, with TM networks enriched for gap junctions, as evidenced by GAP43 and connexin-43 expression, at invasive fronts along with transcriptional enrichment of motility and ECM-remodeling pathways.

Organoid models therefore provide a mechanistic framework linking tumor-neuron signaling to invasion within a human neural context. However, these systems have significant limitations. While they are useful models of neural ECM-tumor crosstalk, these models do not reproduce the biomechanical properties or neurovascular ECM characteristic of the *in vivo* tumor microenvironment. These constraints should be considered when interpreting invasion studies. Advances in tissue engineering that better recapitulate the ECM composition and mechanical properties of tumors are expected to enhance the fidelity and utility of organoid platforms for investigating invasion and identifying new therapeutic strategies to limit tumor spread in the brain.

### 3.3 Organoid models of pediatric gliomas and neurodevelopmental tumorigenesis

Gliomas are the most prevalent histological subtype of brain and central nervous system tumors in children under 14 years, and are a leading cause of cancer-related mortality in children (117). Pediatric gliomas are thought to arise from embryonic and postnatal neural progenitor populations, and are driven by dysregulated neurodevelopmental processes (8, 118, 119). Brain organoids, which can recapitulate aspects of human neurodevelopmental trajectories (23, 120), provide well suited platforms for modeling pediatric gliomas and their interactions with the developing neural microenvironment (**Table 1**; **Figure 2A**).

Benign and low-grade pediatric gliomas (pLGGs) are often slow-growing and difficult to study using conventional tumor cell cultures due to their limited proliferative capacity (121, 122). Organoids systems permit long-term culture and enable modeling of early tumorigenic events during prenatal and early postnatal neurodevelopment. For example, organoids generated from heterozygous *TSC2^+/-^* iPSCs derived from patients with tuberous sclerosis complex (TSC) recapitulate subependymal nodules and cortical tuber–like lesions characteristic of this neurogenetic tumor syndrome (123–125). These models reveal aberrant progenitor expansion, interneuron lineage involvement driven by dysregulated mTOR signaling, and copy-neutral loss of heterozygosity during tumor evolution. They also identify EGFR inhibition as a potential therapeutic strategy beyond mTOR blockade for TSC-associated tumors. Similarly, NF1-mutant cerebral organoids reveal how distinct NF1 mutations disrupt neurogenesis, enhance progenitor proliferation, and delay neuronal differentiation, providing mechanistic insight into the developmental origins of optic gliomas and other pLGGs (126, 127). These studies highlight the utility of organoids for modeling aberrant neurodevelopmental trajectories underlying low-grade pediatric brain tumors.

Organoid systems have also proven instrumental for studying pediatric high-grade gliomas (pHGGs), such as DMGs. For example, engineered hESCs and iPSCs demonstrated that oncogenic histone mutations, including H3.3-G34R and H3-K27M, are permissive but not sufficient for oncogenic transformation on their own, and cooperate with tumor suppressor loss, oncogenic RTK variants, and developmental context to drive malignant pHGG transformation through altered chromatin states and Notch pathway activation (50, 128–130). For example, introduction of H3-K27M mutations into cerebral organoids increases progenitor proliferation, but full malignant transformation requires additional alterations, such as PDGFRA mutant variants and TP53 mutation (42, 50).

Organoid systems have revealed how pHGG cells remodel their microenvironment. For example, patient-derived DMG cells engrafted to microglia- and astrocyte- enriched iPSC-derived brain organoids induce inflammatory and stress-response programs in surrounding neural cells, similar to patient tumors (52, 131). Like adult GBO models, patient-derived pHGG organoids retain genetic, epigenetic, and transcriptional features of their parent tumors (52). Brainstem organoids used to model DMG identify OPC-like cells as candidate cell(s) of origin, validating hypotheses that glial specification acts as a driver of tumorigenesis (8, 42, 132). These platforms also enable identification of tumor-specific vulnerabilities, including responses to clinically relevant targeted therapies, such as ONC201 (52), and evaluation of CAR T-cell therapies within a human neural context (8, 42, 132), supporting their translational relevance (**Table 2**).

## 4 Emerging applications of organoid technologies

Rapid methodological advances are expanding the scope of organoid-based research into new biological and translational domains. In this section, we highlight how vascularization strategies, bioengineering innovations such as microfluidics, and patient-derived platforms are being applied to improve physiologic fidelity and model therapeutic responses.

### 4.1 Vascularized brain organoids for enhancing tumor–neuron interactions

The cerebral vasculature, which forms the blood–brain barrier (BBB), is essential for maintaining neural homeostasis through metabolic support and microvascular interactions with glial and neuronal structures. In gliomas, vasculature influences invasion, with tumor cells migrating along perivascular structures, shapes tumor progression, by providing a tropic niche for GSCs, and modulates therapeutic response through formation of the blood–tumor barrier (BTB) (reviewed in 133, 134). Moreover, neurotrophic factors that mediate tumor-neuron paracrine signaling, such as BDNF (94), also promote tumor angiogenesis by inducing endothelial cell proliferation and migration (135). Thus, neuronal and vascular compartments engage in direct, multi-level functional crosstalk in gliomas. However, the consequences of these interactions on the GBM neural connectome remain to be fully elucidated.

Although self-organizing iPSC-derived brain organoids generate diverse neuronal and glial populations, they lack functional vasculature, resulting in diffusion-limited hypoxia, oxidative stress, and necrotic core formation during prolonged culture (90). These limitations constrain neuronal maturation and limit long-term functional studies. Patient-derived organoid models, such as IPTOs, initially retain tumor-associated vasculature structures and cell types. However, vascular flow is not maintained, and endothelial and perivascular cells progressively decline over time (28), leading to increased hypoxia and reduced microenvironmental complexity.

To address these challenges, multiple strategies are being devised to generate vascularized brain organoids (**Figure 2E**). One approach involves co-culture of iPSC-derived brain organoids with endothelial progenitors or fusion with BBB-like organoids to generate vascularized human cortical organoids (vhCOs) (**Figure 1K**; **Table 1**) (43–45). Addition of VEGF to vhCOs enriches for neurogenesis-associated signatures (43, 45), suggesting that vascular components foster neuronal expansion and differentiation. vhCOs also display endothelial cell networks expressing tight junctions and efflux pumps. Another strategy involves inducing endogenous endothelial cells by overexpressing the endothelial transcription factor ETV2 in a subset of iPSC-derived cells during organoid differentiation (46). In these models, ETV2-driven cells express tight junction components, efflux pumps, and glucose transporters, and demonstrate FITC-dextran perfusion in microfluidic flow assays. However, these endothelial structures do not form multicellular vascular units and are not subject to flow or shear stress in culture. Instead, they are passively perfused by media, and their selectivity to nutrient exchange and barrier function remains poorly understood. Consequently, these models are best considered as simplified, but informative models that represent early steps towards more complex organoid models incorporating additional structural elements and vasculature cellular components that do not yet fully recapitulate functional vascular dynamics.

Continued optimization will be critical to capture metabolic, trophic, and barrier-related vascular interactions relevant to tumor progression, organoid maturation, and neuronal function (**Table 2**). Validation against *in vivo* reference models and primary human tissues will be important for establishing physiological relevance. These efforts will be essential for understanding the broader impact of the neurovasculature on tumorigenesis, which involves more complex cellular networks between tumor cells and neuronal, astrocytes and microglia cells that regulates vascular function in response to neural cues (136).

### 4.2 Bioengineering strategies to improve physiological fidelity of organoid models

Bioengineering strategies are being developed to address key limitations of current organoid systems and to better recapitulate the glioma microenvironment. While existing models capture important aspects of tumor biology, they remain constrained by static culture conditions and the absence of biomechanical cues inherent in the neural microenvironment. Here, we highlight emerging bioengineering strategies to improve glioma organoid platforms.

Mechanical properties of the extracellular environment are key determinants of glioma invasion and recurrence. ECM composition, stiffness, and viscoelasticity influence tumor biomechanics and regulate oncogenic and synaptogenic signaling in primary brain tumors (reviewed in 137, 138). For example, stiffness gradients decrease across the lifespan in the normal brain (139), whereas gliomas exhibit increased stiffness with advancing tumor grade. This shift is driven in part by increased expression and deposition of ECM components, such as hyaluronan and collagens, which promote invasive tumor cell phenotypes within the TME (140–142). Mechano-transduction pathways, including the YAP/TAZ axis, which is a core driver of tumor stem cell identity and diffuse invasion (143, 144), respond to changes in ECM stiffness and cellular geometry and direct tumor invasion along axonal tracts (145, 146), underscoring the role of mechano-transduction in glioma-neuron interactions. Glioma cells also sense mechanical cues via mechanosensitive ion channels such as PIEZO1, which is upregulated in high-grade tumors, and responds to pressure and shear stress to activate Rac-Rho and YAP/TAZ signaling, thereby promoting tumor progression (147, 148). Despite the importance of these pathways, current organoid models do not fully capture these aspects of glioma biology. iPSC-derived cortical organoids exhibit lower stiffness and higher viscoelasticity than human brain tissues and lack ECM comparable to tumors (149). Accordingly, the mechanical properties of organoid platforms should be carefully considered when designing and interpreting studies of glioma migration and invasion.

New culture paradigms offer another route to improve physiologic and biomechanical fidelity. Patient-derived organoid models, such as GBO or IPTO, do not maintain a durable ECM, often requiring supporting scaffolds such as Matrigel (150). Incorporation of ECM-like materials that more closely represent tumor ECM, with defined matrix components benchmarked against primary tissues, will be necessary to improve physiological relevance. Synthetic and tunable biomaterials, such as engineered hydrogels with adjustable stiffness, viscoelastic relaxation, and ligand presentation, have emerged as platforms to decouple biochemical from biomechanical signaling. For example, stress-relaxing hydrogels regulate stem cell fate through viscoelasticity, and studies of 3D hydrogel models of patient-derived GBM demonstrate that matrix stiffness modulates proliferation, invasion, and drug resistance (141, 151).

Microfluidic strategies are also emerging as powerful tools for introducing controlled biomechanical forces that are difficult to reproduce in static culture systems. These approaches allow mechanistic investigation of how physical forces regulate tumor cell migration and invasion and multicellular interactions within defined microenvironments (152–154). To more precisely control perfusion, nutrient gradients, and immune cell interactions *in vitro*, organ-on-a-chip platforms have been developed (**Figure 1J**). These micro-engineered microfluidic systems are designed to recapitulate key physiological and structural features of specific tissues (155). By seeding pluripotent stem cells or tissue-derived cells within microfabricated vessels, investigators can manipulate defined aspects of the cells and their microenvironment to model organ or tissue-specific conditions (**Table 1**). Organoid-on-chip platforms enable regulation of shear stress, perfusion, electrical, mechanical, and chemical stimuli, as well as physical parameters such as organoid size and geometry (47). Perfusion systems mimicking vascular flow can alleviate hypoxic stress and diffusion limitations and facilitate modeling of BBB penetration by chemotherapies and nanoparticles in endothelial–pericyte–astrocyte co-culture systems incorporating GBM cells (156–162) (**Table 2**). Pump-controlled flow reduces waste accumulation and more closely reproduces shear stress and flow directionality, which can be monitored in real-time by live imaging approaches (163). In addition, microgravity based systems using continuous rotation can improve oxygen and nutrient diffusion, enhance organoid growth and viability beyond conventional culture conditions, and enable controlled delivery of small molecules and therapeutics (164), including CAR T-cells.

These bioengineering approaches represent important advances toward improving the physiological relevance of glioma organoid models. By incorporating tunable ECM components, controlled mechanical forces, and perfusion, these systems can address key limitations of conventional static organoid cultures. However, challenges remain, including incomplete representation of immune and vascular components of the TME and the lack of physiologic perfusion required for proper waste exchange and tissue homeostasis. Continued development of integrated platforms combining biomaterials, microfluidics, and multi-organoid assembloid systems is likely to enhance model fidelity and expand their utility for studying glioma biology and therapeutic response. As these technologies mature, they have the potential to serve as predictive platforms for translational mechanistic studies and precision medicine applications.

### 4.3 Organoid models and modeling brain tumor therapeutic responses

High-grade gliomas, including GBM, show profound resistance to current standard-of-care (**Figure 2F**). This resistance arises, in part, from extensive intrinsic molecular and cellular heterogeneity, and is reinforced by extrinsic, protective features of the neural tumor microenvironment (165). As organoid technologies advance, they are increasingly positioned as platforms for therapeutic discovery, response prediction, and mechanistic dissection of treatment response and resistance in both adult and pediatric neuro-oncology.

Patient-derived GBM organoids, including GBOs (28), have been adapted to evaluate chemotherapy, radiotherapy, DNA-damaging agents, targeted agents, and immunotherapy (166). These models have been used to evaluate responses to standard-of-care treatments, such as Temozolomide (TMZ) chemotherapy, and radiotherapy, benchmarked to clinical outcomes (28, 51, 166). For example, treatment of GBOs with agents including vemurafenib, trametinib, and TMZ reveals heterogeneous, patient-matched sensitivities that correlate with tumor-specific driver mutations and clinical radiologic responses (167), reflecting interpatient heterogeneity. GBOs also retain endogenous expression of clinically relevant antigens, including EGFR variants (28), enabling evaluation of targeted therapies. Co-culture with CAR T-cells demonstrates antigen-specific infiltration, immune activation, cytokine secretion, and tumor cell killing, underscoring the value of GBOs for immunotherapy development (28, 168). Despite their advantages, GBOs can be highly variable and incompletely recapitulate the TME. To address these limitations, alternative models have been developed. The iHOTT model incorporates patient-derived tumor cells and matched peripheral blood mononuclear cells (PBMCs) within iPSC-derived cortical organoids, introducing an autologous human immune compartment (**Figure 1E**) (54). After one week of co-culture, the immune population is primarily composed of circulating lymphoid cell types, including T and B cells, with limited representation of myeloid populations (**Table 2**), reflecting challenges in recreating tissue-resident and infiltrative innate immune lineages (17). Therefore, results from these systems require interpretation in reference to their incomplete TME representation.

More complex patient-derived models that retain both neural and immune elements can offer further insight. IPTOs retain myeloid cells, T-cells, and vascular cells, and have been used to model patient-specific treatment responses (51). For example, molecular signatures of recurrence in TMZ-treated IPTOs closely matched clinical outcomes: following TMZ exposure, NPC-like tumor states are depleted, whereas proliferative AC-like and MES-like populations expand, implicating these states in TMZ resistance, consistent with observations from matched primary and recurrent tumor specimens (5). Tumor-associated macrophages also exhibit TMZ-associated resistance signatures, and combined TMZ and CSF1R inhibition disrupts macrophage–tumor interactions and reveals metabolic dependencies that emerge upon treatment pressure (51). However, these systems remain limited by the finite maintenance of immune and stromal components, which typically persist for 2–8 weeks in culture.

Patient-derived organoid platforms have also been applied across adult and pediatric gliomas to evaluate patient-specific therapeutic responses (28, 52, 169). For example, screening across patient-derived glioma organoid cohorts and across tumors from distinct molecular subtypes and subregions, including IDH-mutant and H3-K27M tumors (170), revealed marked variability in responses to kinase inhibitors, underscoring the utility of organoids for evaluating the contributions of genetic and spatial heterogeneity to treatment response. Integration of patient-derived organoid models with molecular profiling and functional readouts, such as viability assays, live imaging, and single-cell analyses, enables mechanistic investigations of therapeutic response and resistance (28, 79, 166). Nonetheless, broader application of these approaches to drug discovery remains constrained by access to patient material, lack of assay standardization, and absence of physiologic vascular-BTB components predictive of drug bioavailability.

Glioma organoids are particularly valuable for interrogating therapeutic vulnerabilities arising from tumor–neuron communication. For example, GBOs retain functional tumor–neuron synapses and express GABA_A receptor subunits, particularly GABA_Aα1 and GABA_Aγ1 (171). Consistent with evidence that gliomas integrate into GABAergic neural circuits that stimulate tumor growth and progression (171, 172), pharmacologic inhibition of GABAergic signaling using GABA_A antagonists significantly reduces tumor proliferation and invasion in GBOs (171). This demonstrates that organoid models can serve as predictive platforms for therapies that disrupt synaptic dependencies. iPSC-derived organoid models extend these capabilities by facilitating direct investigation of tumor invasion and electrochemical coupling with neurons. Cerebral organoid-glioma co-culture models show that GBM cells form active gap junctions with neurons and other tumor cells, recapitulating *in vivo* connectivity (29). Pharmacologic inhibition of tumor–neuron calcium signaling, by blockade of gap junctions with meclofenamic acid or inhibition of KCa3.1 channels, suppresses tumor proliferation and progression (100). Complementary studies on GBOs demonstrate that connexins Cx26, Cx30, and Cx32 contribute to glioma invasion and neuronal coupling, and that therapeutic blockade of these gap junction components impairs tumor progression (173, 174). Organoids can also be used to evaluate neurotoxicity. For example, high-throughput screening of iPSC-derived glioma assembloids identified compounds that suppress glioma infiltration without overt neuronal toxicity (175). Together, these studies indicate that such models can be used to simultaneously assess therapeutic efficacy and potential adverse effects on neural circuits.

Despite their promise, both iPSC-derived brain organoids and patient-derived glioma organoids present technical caveats for therapeutic discovery. Significant challenges include access to patient-derived materials, variability across models, incomplete representation of immune and stromal components, and assay complexity and turnaround time (176). Additional complications include lack of physiologic vascular-BTB components, diffusion-limited nutrient and oxygen gradients, and hypoxia-related stress within organoid cores, which complicate modeling of drug delivery and pharmacokinetics. Efforts to address these challenges include development of standardized, high-throughput organoid systems. For example, spinning bioreactor-based iPSC-derived brain organoid cultures, which reduce variability and cellular stress while improving cytoarchitectural consistency (175), can support robust invasion by patient-derived GBM cells to enable scalable drug screening, and were used to identify compounds such as fulvestrant and selumetinib as inhibitors of invasion (177, 178).

In summary, organoid-based platforms, particularly patient-derived glioma organoids, have emerged as powerful translational models for studying therapeutic responses in neuro-oncology. By preserving patient-specific heterogeneity and facilitating interrogation of dynamic tumor–neuron interactions within a human neural context, these systems provide insight into mechanisms of treatment response, resistance, and recurrence. They have revealed therapeutic vulnerabilities linked to neural activity, synaptic signaling, and microenvironmental coupling that are difficult to capture in conventional culture systems. However, their broader application for drug discovery remains constrained by challenges with scalability and standardization. Continued methodological refinement and integration with complementary model systems will be essential to expand their translational utility and capitalize on their potential in precision medicine.

## 5 Current and future challenges in organoid-based modeling of the glioma microenvironment

Despite the rapid expansion of organoid-based platforms for modeling glioma biology, several technical and biological challenges remain that affect microenvironmental fidelity, physiological relevance, and experimental reproducibility.

A central limitation is the incomplete representation of key components of the tumor microenvironment. As previously discussed, current organoid models lack functional neurovasculature and durable vasculature-generated ECM, which are critical for tumor progression and therapeutic response. In addition, tissue mechanics and biomechanical signaling, important elements of brain tumor biology, are not adequately addressed. Future studies will need to incorporate vascular elements, integrate mechano-signaling pathways, and preserve innate immune populations to define how microenvironment cues shape invasion modes and modulate tumor-neuron interactions. Emerging approaches, such as microfluidic devices, remain under development to provide perfusion and improve nutrient delivery, promoting greater physiologic fidelity. Moreover, other cellular and structural elements in the human brain, such as myelinated nerve fibers characteristic of white matter tracks that also serve as substrates for tumor invasion, are not represented in current tumor organoid models.

Another major challenge is ensuring that organoid health and structural integrity do not confound analyses of invasion, metabolism, or therapeutic response. 3D cultures are inherently sensitive to culture conditions such that compromised baseline viability can distort treatment effects or alter tumor cell-state distributions. The development and implementation of rigorous systematic quality-control practices represent an important methodological priority that can strengthen reproducibility across studies. Conventional approaches, including histologic assessment and immunostaining for tumor subtype markers, proliferation, and microenvironmental composition, provide robust validation but are often destructive and applied retrospectively, limiting their utility for prospective experimental application. These and many other current quality control practices, however, can be inherently destructive, which precludes more rigorous prospective quality control.

Strategies that preserve organoid integrity will be essential for improved experimental rigor. For example, longitudinal brightfield imaging of organoid morphology enables automated or high-throughput microscopy monitoring methods. Healthy organoids typically exhibit a bright white-cream color, smooth, rounded refractive edges, compact cellular density, and a solid structure, whereas compromised or necrotic organoids may adopt a darker discolored tan appearance, lose edge integrity, and shed cells and debris. Growth kinetics, including consistent size progression and proliferative activity, provide additional indicators of viability. Media-based readouts, such as color changes reflecting metabolic or reporter activity, can also be assessed. These approaches are suited to robust, non-destructive quality control, ensuring that experimental outcomes accurately reflect tumor biology rather than variability in organoid health.

Several strategies are emerging to address developmental and microenvironmental limitations. Prolonged maturation protocols and air–liquid interface cultures enhance neuronal survival, synaptic integration, and astrocytic complexity (49), partially narrowing the developmental gap between organoids and mature brain tissue. Transcriptional benchmarking against human reference atlases permits quantitative staging of organoids, allowing investigators to contextualize therapeutic findings relative to developmental identity. Efforts to incorporate immune cells, vascular components, and engineered ECM matrices into brain organoids are underway to improve microenvironmental fidelity (30, 53, 54). Parallel comparative experiments across platforms, including iPSC-derived assembloids and patient-derived tumor organoids such as IPTOs (51), provide a framework to disentangle tumor-intrinsic mechanisms from developmental-context effects.

Taken together, iPSC-derived organoid systems are best interpreted as controlled human neural platforms for mechanistic dissection and genotype-specific interrogation, particularly of neurodevelopmental processes and neuron-tumor interactions. In contrast, patient-derived tumor organoid systems retain native tumor structure, genetic heterogeneity, and elements of the tumor microenvironment, making them valuable for studying patient-specific tumor evolution and therapeutic response. Integrating insights from these complementary systems therefore provides a more comprehensive framework for investigating glioma biology across developmental and microenvironmental contexts. However, rigorous interpretation requires explicit consideration of developmental stage, benchmarking strategies, and validation in independent model systems.

## 6 Discussion and summary

High-grade gliomas, including GBM and DMG, comprise a heterogenous group of lethal brain tumors that remain largely refractory to current therapies. Despite aggressive multimodal treatment, recurrence is almost inevitable and is associated with lethal outcomes. A central challenge in neuro-oncology is therefore the need to more faithfully model the biological features of these tumors that drive therapeutic resistance, recurrence, and neurological dysfunction, with the goal of advancing the discovery of new treatment strategies to improve patient outcomes and quality of life.

In recent years, a diverse array of three-dimensional organoid-based platforms has emerged to address this need by modeling glioma biology within a human neural context. These models mark a conceptual shift in neuro-oncology away from tumor-intrinsic paradigms toward model systems that incorporate key features of the human neural tumor microenvironment. Compared with traditional tumor cell monoculture culture models, organoid platforms better recapitulate tumor spatial organization, multicellular interactions, and context-dependent tumor states central to glioma biology, including genetic and epigenetic heterogeneity, tumor-neuron synaptic integration, regionally patterned invasion and circuit integration, and hypoxia-structured GSC niches. Moreover, these models can be augmented with immune and vascular components, further enhancing biological relevance.

An important consideration in interpreting organoid studies is their developmental identity. Most iPSC-derived cerebral organoids transcriptionally and epigenetically resemble embryonic or early developmental cortex, and therefore do not adequately model the aging adult brain in which GBM typically arises. Aging strongly shapes the neural microenvironment, yet organoid models generally lack key characteristics of the aging brain, such as chronic inflammation, glia remodeling, vascular aging, and senescence-associated signaling (14, 179). That said, GBM frequently reactivates developmental regulatory programs, reverting or re-enforcing immature cell states that are maintained in tumor cells engrafted into iPSC-derived models. This likely reflects inductive interactions between tumor cells and neurons and glia within the developmentally permissive niche of neural organoids. Addressing this mismatch will require new strategies that reproduce aspects of the aged brain, supported by rigorous benchmarking against adult reference datasets and incorporation of adult-derived stromal and immune components, to better approximate the aged neural niche.

A major strength of organoid-based glioma models lies in their ability to capture human-specific aspects of tumor–neuron interactions that are difficult to study in animal models due to species specific divergences in neurodevelopment, neural circuitry, and cellular composition. Organoids enable interrogation of human specific aspects of neuron-to-glioma signaling, glial lineage programs, and regional neural niches that shape tumor cell states and therapeutic vulnerabilities. These platforms are also amenable to direct experimental perturbation, including CRISPR editing, pharmacologic modulation, lineage tracing, and synaptic mapping, while preserving key features of tumor heterogeneity. This flexibility permits longitudinal and dose-dependent mechanistic studies that are often impractical in animal models. Importantly, patient-derived organoid systems allow relatively rapid functional testing of potential therapies on human tumor tissues, facilitating hypothesis-driven evaluation of combination therapies, including targeted agents and emerging immunotherapies.

Despite their promise, organoid systems remain constrained by important biological and technical limitations. iPSC-derived brain organoids lack a functional vasculature and fail to recreate BBB or BTB function, limiting their suitability for assessing bioavailability of drugs of other therapeutics. They also incompletely represent immune populations, particularly tissue-resident and infiltrating myeloid cells, which play critical roles in glioma progression and therapy response. Patient-derived organoids retain greater microenvironmental complexity but exhibit progressive loss of neural, vascular, and immune dynamics over time and are limited by tissue availability and inter-tumoral heterogeneity. Technical challenges currently limit the reproducibility and scalability of organoid models. These include batch-to-batch variability, differences in maturation state, and the requirement for specialized expertise in iPSC cell engineering and organoid culture. Across both models, standardization of culture conditions, analytical pipelines, and readouts remains incomplete, and experiments are time- and resource-intensive, requiring specialized expertise in imaging and multi-omics. These constraints argue against viewing organoids as replacements for animal models. Instead, their value lies in serving as complementary human-specific discovery and validation engines that can prioritize hypotheses, mechanisms, and therapeutic strategies for further *in vivo* validation and clinical translation.

Importantly, no single organoid platform is universally optimal. Model selection should be guided by specific biological question under investigation. iPSC-derived organoids offer experimental control and scalability, patient-derived organoids maximize clinical fidelity but are limited by access to patient-derived tissues, and organotypic slice cultures preserve native architecture but are constrained in longevity and throughput. All models remain simplifications of the human brain tumor microenvironment, their limitations should be considered in experimental design and interpretation. Thoughtful integration of complementary experimental models is essential for rigorous investigation.

Impactful progress is likely to arise from refinement and standardization of existing organoid technologies rather than expansion of new formats. Key priorities include more consistent incorporation and preservation tissue components that govern treatment failure in patients, including integration of functional neural circuitry. Advances in bioengineering, including microfluidics and assembloid methodologies, promise to improve perfusion, reduce necrosis, and facilitate controlled delivery of therapeutics. Improvements in scalability, reproducibility, and throughput will facilitate broader adoption of organoids for mechanistic discovery pipelines and precision medicine applications. As these technologies mature, glioma organoids are poised to evolve from powerful experimental models into decision-shaping translational platforms and to accelerate the development of effective, patient-informed therapies.
